# Influence of back extensor strength on the natural history of kyphosis

**DOI:** 10.1186/1748-7161-10-S1-O27

**Published:** 2015-01-19

**Authors:** Michio Hongo, Naoshisa Miyakoshi, Yuji Kasukawa, Yoshinori Ishikawa, Daisuke Kudo, Yoichi Shimada

**Affiliations:** 1 Department of Orthopedic Surgery, Akita University Graduate School of Medicine, Japan

## Introduction

Previous cross-sectional study demonstrated that spinal kyphosis was signiflcantly associated with back extensor strength. However, the influence of trunk muscle on the development of kyphosis is still unclear due to the paucity of longitudinal study. The purpose of this study was to evaluate the association of back extensor strength and progression of kyphosis with longitudinal follow up.

## Methods

Thirty women with an average age of 66 years were included in the study. They were followed up for more than two years. Participants who had severe kyphosis with 2 or more vertebral fractures or recent fracture within 6 months and who had underwent back strengthening exercise were excluded. Thoracic kyphosis, thoraco-lumbar, lumbar lordosis, pelvic tilt, sacral slope, pelvic incidence, and sagittal vertical axis were measured with lateral standing radiographs of whole spine including pelvis at the baseline and final follow-up. Isometric back extensor strength and grip strength were also measured with dynamometer.

## Results

Significant changes were found in sagittal vertical axis (13.9mm to 24.8mm, p=0.008) and thoracolumbar kyphosis (7.5 degrees to 9.8 degrees, p=0.02). Whereas, there was no significant change in the measurements for thoracic kyphosis, lumbar lordosis, pelvic tilt, pelvic incidence, sacral slope, back extensor strength, and grip strength at the final follow up. Then analyses were performed to evaluate the factors contributing to the increase in sagittal vertical axis. The change in sagittal vertical axis was significantly correlated with age, grip strength, and back extensor strength, but not with other radiographic spino-pelvic measurements.

## Conclusion

Increase in sagittal vertical axis was significantly associated with back extensor strength and grip strength, irrespective of spin-pelvic alignment, indicating the importance of strengthening the physical performance including back extensor strength for preventing the development of spinal kyphosis.

